# Investigation of Electromagnetic Shielding and Flame Retardancy Properties of Thermosetting-Based Hybrid Composites Containing Fe_3_O_4_ and Activated Carbon Obtained from Buckwheat Hulls Waste

**DOI:** 10.3390/polym18172121

**Published:** 2026-08-31

**Authors:** Akın Odabaşı, Essam Bkkur

**Affiliations:** 1 Engineering Faculty, Department of Metallurgical and Materials Engineering, Fırat University, 23200 Elazığ, Turkey; 2 Graduate School of Natural and Applied Sciences, Metallurgical and Materials Engineering, Fırat University, 23200 Elazığ, Turkey; essambakkur.swi@gmail.com

**Keywords:** flame retardant, EMI shielding, novolac, activated carbon, Fe_3_O_4_ particles, limiting oxygen index

## Abstract

The demand for materials with flame-retardant and electromagnetic shielding properties has increased research on sustainable and multifunctional composites. Thermoset-based hybrid composites containing Fe_3_O_4_ and activated carbon obtained from solid waste were investigated for flame retardancy and electromagnetic shielding. Activated carbon produced by pyrolysis of buckwheat hulls (agricultural waste) exhibited a BET surface area of 714.33 m^2^/g and a conductivity of 69.5 S/m, and was used as a filler to enhance electrical conductivity, while Fe_3_O_4_ was added to promote electromagnetic absorption. Composites with 1, 3, 5 and 7 wt% activated carbon at a constant 15 wt% Fe_3_O_4_, together with a complementary series at 5 wt% activated carbon with 10, 15, 30 and 45 wt% magnetite, were characterized by limiting oxygen index and thermal analysis. LOI values clustered between 32.31% (Nov-5-15) and 33.71% (Nov-3-15), a 1.40-percentage-point spread around the 34.31% reference Novolac. The 850 °C char yield peaked at 56.38% (Nov-3-15) and 55.34% (Nov-7-15), with T_50_% reaching 898 °C and 882 °C, respectively, while DTA replaced the 529 °C Novolac exotherm with endothermic Tmax values of 485–501 °C. Electromagnetic shielding effectiveness over the 8–12 GHz range varied from 2.97 ± 0.3 dB (unfilled Nov-0) to a maximum of 9.19 ± 1.46 dB (Nov-5-45, 5 wt% activated carbon and 45 wt% Fe_3_O_4_), with an intermediate value of 7.43 dB for Nov-7-15. These hybrids are thus candidate materials for fire-safe phenolic-thermoset applications, where magnetic–dielectric coupling and a percolated char-barrier network govern flame-retardant performance, demonstrating the potential of waste-sourced carbon in sustainable composite production.

## 1. Introduction

Polymer-based composite shielding materials for electromagnetic interference (EMI) protection have attracted considerable research attention due to their significant advantages of light weight, adjustable conductivity, processability, and corrosion resistance [1,2]. Based on the principle of electromagnetic interference shielding, many complex factors have been found to play a significant role in achieving the desired shielding efficiency for conductive polymer composites. First, the type, morphology, quantity, and distribution of particles significantly influence the properties of conductive polymer composites [2,3,4]. Surface conductive layers or products with high metal content provide good electromagnetic interference shielding primarily through reflection, but they can often lead to high reflection, resulting in strong secondary electromagnetic radiation [5,6]. Therefore, reflection-based EMI shielding can only prevent interference with the shielding material but cannot effectively control and eliminate lost electromagnetic waves [6,7,8]. Therefore, eliminating secondary electromagnetic wave pollution can be achieved by adding magnetic particles and carbon-based hybrid materials (such as RGO, graphene, and MWCNT) to enhance the absorption properties of conductive polymer composites [9,10]. Here, magnetic particles provide magnetic loss, while carbon-based fillers contribute to dielectric loss, which can scatter electromagnetic waves more effectively through absorption [11].

Improving flame retardancy in polymer composites is a critical area for safety and performance. Properties such as thermal stability, low flammability, and high charring rates of flame retardant polymer composites enable them to withstand the high temperatures encountered in fires. Fire resistance is enhanced, particularly in flammable polymer matrices such as thermoset resins, through the use of various fillers and additives such as alumina trihydrate, magnesium hydroxide, or silica [12]. In recent years, flame-retardant nanomaterials and hybrid composites have gained prominence, offering both environmental friendliness and high efficiency [13,14]. Investigating the effect of carbonaceous fillers on the fire performance of polymeric materials is very important because fillers with different carbon sources, such as carbon black, graphite, graphene and carbon nanotubes, have been widely used to improve the electrical and mechanical properties of polymer matrices [15].

Liu et al. [16] developed nanocomposites containing nanosized multi-walled carbon nanotubes (MWCNTs), Fe_3_O_4_, and an Fe-filled epoxy matrix for electromagnetic interference (EMI) shielding applications. Furthermore, three-layer laminated nanocomposites were designed and fabricated, consisting of a matching layer containing 15 wt% nano-Fe_3_O_4_, an absorber layer containing 5 wt% CNTs, and a reflector layer containing 10 wt% CNTs. Experimental results showed that such three-layer laminated nanocomposites possess excellent microwave absorption effect in the frequency band (13–40 GHz), up to 40 dB [16].

Joon et al. produced lightweight and processable thin poly(o-toluidine)-carbon fiber (PoTCF) composite sheets for electromagnetic interference shielding. The composite material, prepared by physically blending the PoTCF composite with novolac resin at different weight ratios, exhibited a shielding effectiveness of 24 dB in the X-band (8.2–12.4 GHz) when containing 50% novolac resin by weight [17]. Gogoi et al. prepared composite pellets by mechanically mixing novolac phenolic resin as the base matrix and expanded graphite at different weight ratios. The composite containing 50 wt% expanded graphite exhibited a maximum electrical conductivity of approximately 147 S/cm and a reflection loss of >−13 dB along the X-band [18]. In another study, using expanded graphite and novolac resin, Kocaman et al. investigated the mechanical, thermal, and flammability properties of composites prepared by adding varying amounts of expanded graphite to powdered novolac resin. The highest % LOI value of 40.64% was achieved in the composite sample containing 20% expanded graphite [19].

The aim of this study is to investigate the electromagnetic shielding and flame retardancy properties of Novolac-type thermoset composites containing magnetite (Fe_3_O_4_), which is produced by the hydrothermal method from iron salts and activated carbon obtained from buckwheat hulls, an agricultural waste. The microstructure–property relationships of the hybrid composites will be investigated by considering the amount, morphology, and distribution of fillers that can affect EMI efficiency. This will contribute to the studies in the field of thermoset composites that can be used as lightweight and flexible EMI shielding materials.

## 2. Experimental Section

### 2.1. Activated Carbon Production

**Figure 1:**
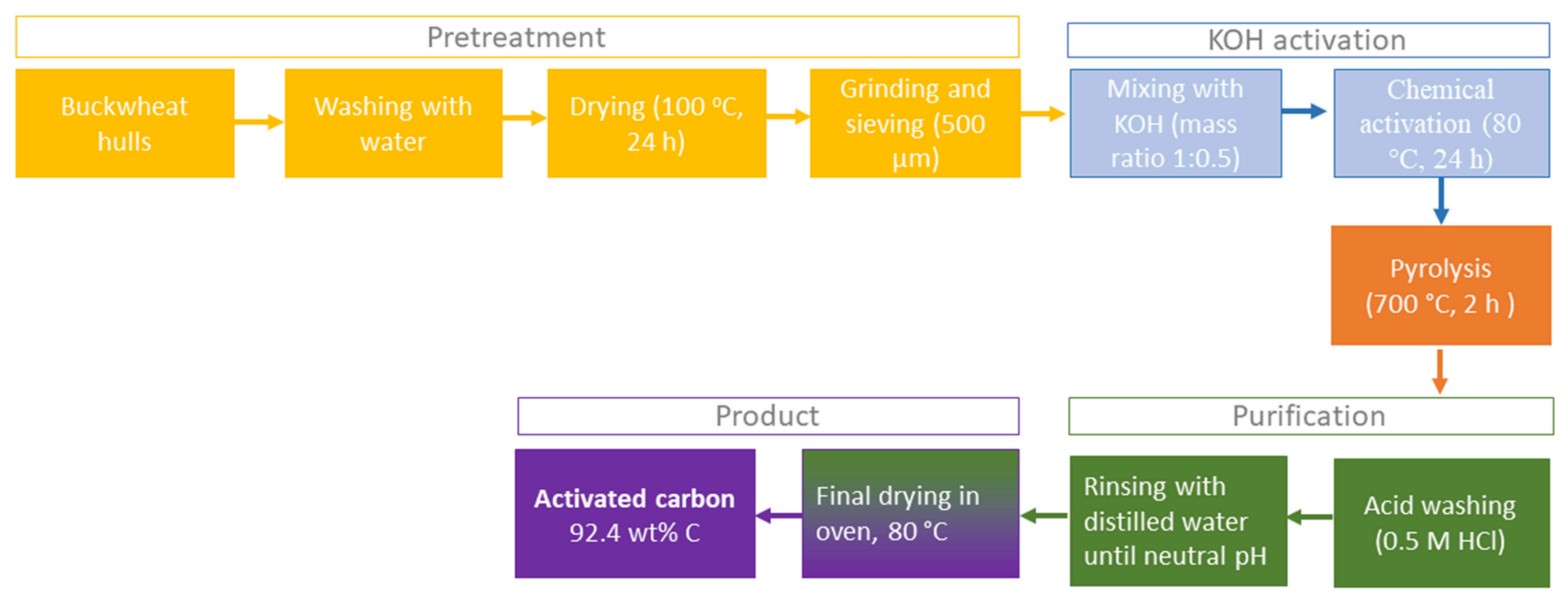
Schematic representation of the activated-carbon production process from buckwheat hulls.

In this study, activated carbon was synthesized by chemical activation and pyrolysis of buckwheat hulls. Figure 1 gives the schematic representation of the activated carbon synthesized from buckwheat hulls. First, the buckwheat hulls were washed with water to remove foreign matter. Buckwheat hulls were dried in an oven at 100 °C for 24 h. The dried material was mechanically ground and sieved through a 500 µm sieve to obtain a powder. The obtained buckwheat hull powder was mixed with potassium hydroxide (KOH) at a mass ratio of 1:0.5, and the prepared mixtures were dissolved in 80 mL of pure water. The resulting mixtures were kept in an oven at 80 °C for 24 h to allow chemical activation. When buckwheat hulls are mixed with KOH (potassium hydroxide) and water is added, an alkaline extraction process essentially takes place. In this case, KOH can dissolve and degrade lignin, particularly in alkaline environments, resulting in the cleavage of aromatic rings and the release of phenolic compounds. Hemicelluloses also become soluble in alkaline environments. Cellulose is quite resistant to alkali. However, under high temperatures and prolonged contact, it may partially swell or begin to dissolve. Oxidation of the resulting phenolic compounds produces a dark brown-black solution. The activated mixture was pyrolyzed in a muffle furnace at 700 °C for 2 h under an N_2_ environment as a protective gas. The carbonaceous structure obtained after pyrolysis was washed with 1 L of 0.5 M hydrochloric acid (HCl) solution to remove inorganic residue. Following the acidic wash, the sample was rinsed with distilled water, and the washing process continued until the pH value reached neutrality. In the final stage, the resulting activated carbon sample was dried in an oven at 80 °C and prepared for analysis.

To determine the surface properties of the obtained activated carbon, a BET (Brunauer, Emmett, and Teller) analysis sample was taken, and to determine the crystal structure, an X-ray diffraction sample was taken, and an electron microscope (SEM) sample to learn its structural and morphological properties, and the remaining part was kept under vacuum until it was used as an additive in composite production. Scanning electron microscope examinations were performed using a FIB-SEM (Thermofisher Scientific Scios 2, Waltham, MA, USA) instrument. The sample was then subjected to phase analysis using an XRD (Rigaku MiniFlex 600, Rigaku Corporation, MiniFlex 600, Tokyo, Japan) device at a scanning speed of 2°/min with a step size of 0.02°. Additionally, the activated carbon sample was subjected to BET surface area analysis using a Micromeritics TriStar II Plus model device (Micromeritics Instrument Corporation, Norcross, GA, USA) and conductivity tests using an Entek Electronics Four Point Probe (Entek electronic, Ankara, Turkey), and the results were recorded.

### 2.2. Production of Fe_3_O_4_ Particles

Fe_3_O_4_ particles were synthesized using the solvothermal method. In this context, a homogeneous solution was prepared by dissolving 10.4 g of FeCl_3_·6H_2_O in 2 g of sodium citrate (Na_3_C_6_H_5_O_7_), 16 g of sodium acetate (NaOAc), and 160 mL of ethylene glycol (EG). The prepared solution was stirred on a magnetic stirrer for 2 h to ensure homogeneity. The mixture was then transferred to an autoclave reactor with a Teflon chamber, and solvothermal treatment was applied at 200 °C for 10 h. The resulting black-coloured Fe_3_O_4_ particles were centrifuged and washed sequentially with acetone and ethanol. Finally, the sample was dried in a vacuum oven at 80 °C for 12 h before being made ready for use. As in activated carbon, FIB-SEM and XRD analyses were carried out to determine the structural and morphological properties of Fe_3_O_4_ particles.

### 2.3. Production of Hybrid Composites

**Figure 2:**
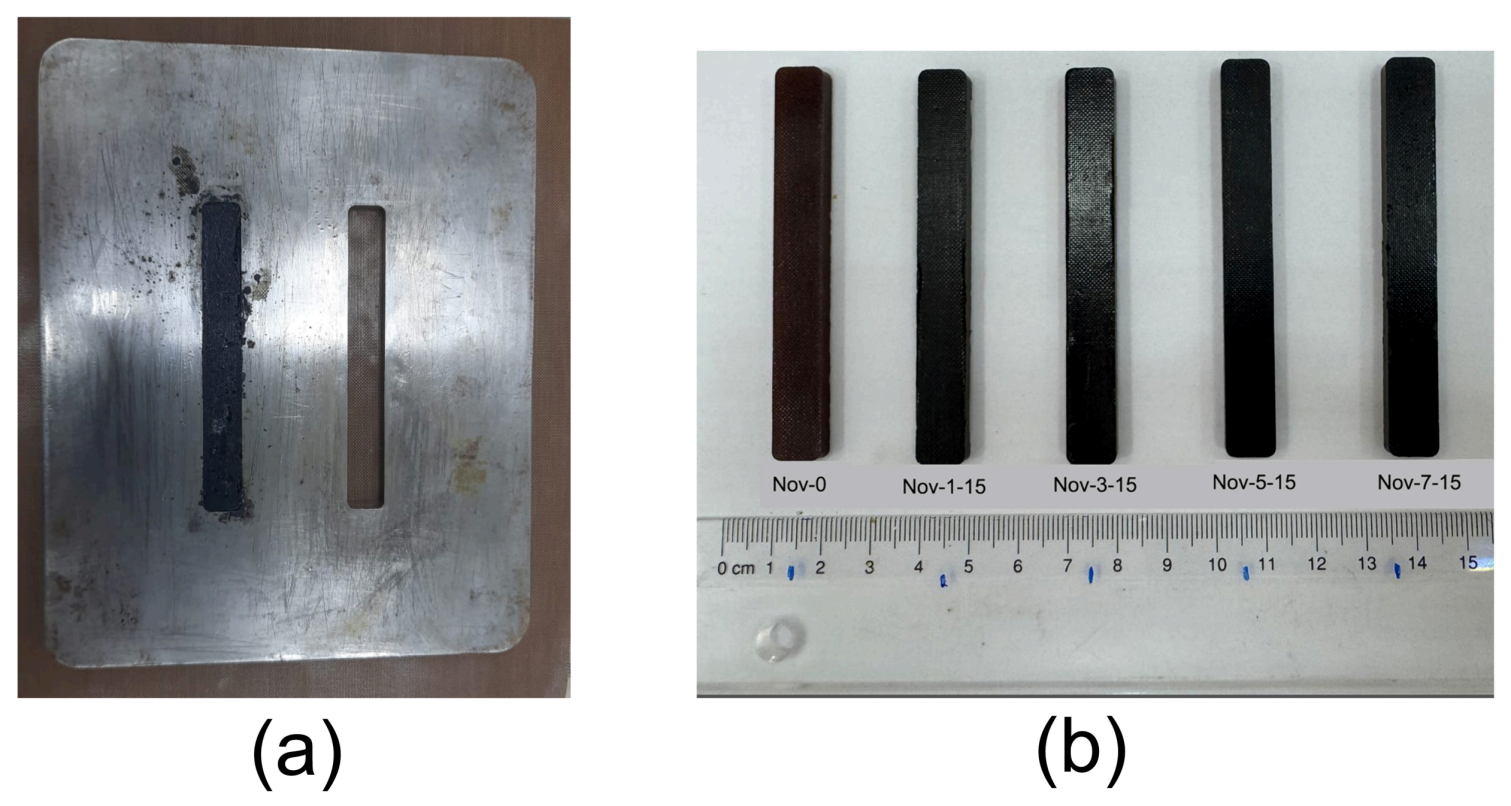
(**a**) Steel mold used for specimen preparation and (**b**) prepared composite samples with different weight percentages of activated carbon and Fe_3_O_4_ particles.

The thermosetting matrix phase used in this study was selected as powdered Novolac resin. Novolac resin is a polymer obtained by the condensation of phenol and formaldehyde in an acidic catalyst environment. It is initially thermoplastic but acquires thermosetting properties under appropriate crosslinking and heating conditions. Thanks to its powder form, it can be easily mixed homogeneously with fillers of different proportions (activated carbon, Fe_3_O_4_) and then a curing process is applied to create a composite structure. In this study, Novolac resin was supplied by Daussan Refrakter company (Manisa, Turkey). Activated carbon, Fe_3_O_4_, and Novolac resin, weighed in the proportions given in Table 1 and Table 2, were mixed in a mixer for 2 h to obtain a homogeneous mixture. The fabrication process was conducted in two stages. Initially, a preliminary group of samples was prepared to evaluate the baseline EMI shielding effectiveness. Based on the initial EMI SE performance of Group 1, the filler loadings for the second group were determined to optimize the shielding properties. The detailed explanation was given in Section 3.8. Table 1 and Table 2 show that the compositions of hybrid composite samples were produced in two different groups. In the first group, the amount of Fe_3_O_4_ was kept constant at 15wt.%, and in the other group, the amount of activated carbon was kept constant at 5 wt.%. The prepared mixture was poured into sample cavities in metal molds, manually compacted, and preformed. It was then pressed in a hot press machine at 150 bar and 160 °C for 30 min. After the pressing process was completed, the samples were cooled in the mold (Figure 2a,b) and then removed from the mold. Thus, samples with dimensions of 80 × 10 × 4 mm were obtained (Figure 2b).

### 2.4. Measurements

In this study, various instruments were used to determine the structural, morphological, thermal, and electromagnetic shielding properties of the fabricated composite materials. The fracture surface morphology and structure of the composite samples were examined using scanning electron microscopy (FIB-SEM), and energy dispersive spectroscopy (EDS) was used to determine their elemental composition. Evaluation of thermal degradation behavior was carried out with a thermogravimetric analysis (Shimadzu DTG-60 TG/DTA, Shimadzu Corporation, Kyoto, Japan) device, and the Limit Oxygen Index (LOI) values of the first and second group samples were calculated using the following formula for the char amounts at 850 °C obtained from differential thermal analysis [19,20,21].

LOI or OI = 17.5 + 0.4µ(1)

In the 1970s, Krevelen revealed the importance of char yield (μ or CR) by reporting the linear relationship between the μ/wt% of pure halogen-free polymers measured by thermogravimetry under nitrogen and the fire characteristic oxygen index (OI). In the formula, μ represents the ratio of the char amount [21]. Additionally, to discuss the reliability of the LOI values obtained from the char residue amount and LOI calculations using the relevant formula, LOI tests on the first group of composite samples were conducted using a Concept brand LOI tester in accordance with ASTM D 2863 [22] and ISO 4589-2 standards [23]. The test method is based on observing the combustion behavior of the sample in a controlled oxygen/nitrogen gas mixture atmosphere using an overhead flame. The samples were fixed in a vertical position, and the flame was determined to be self-sustaining during the test. The oxygen percentage was gradually reduced to determine the lowest oxygen concentration at which the sample could sustain combustion, and this value was expressed as % LOI by volume.

XRD pattern was recorded with a Rigaku MiniFlex 600 diffractometer (Rigaku Corporation, Tokyo, Japan) with Cu radiation (40 kV and 15 mA) using a step-scanning method (Scanning Speed: 2°/min, Step Width: 0.02°) between 2θ = 5–90° to measure the carbon 002 diffraction peak.

TGA/DTA studies were conducted to evaluate the thermal stability and heat capacity of hybrid composite samples. These analyses allow us to determine the thermal degradation of composites, stepwise decomposition processes, and important thermal parameters such as glass transition temperature (Tg) and melting temperature (Tm). TGA/DTA analysis was carried out in the temperature range of 30–900 °C at a rate of 10 °C/min in an ambient atmosphere. The EMI shielding effectiveness (SE) in the frequency range of 8–12 GHz (X-band) was measured using a vector network analyzer (VNA) connected to a rectangular waveguide. Using this method, the samples were evaluated for their reflection, absorption, and total shielding performance against electromagnetic waves. Reflection loss (SE_R_), absorption loss (SE_A_), and total shielding effectiveness (SE_T_) were calculated using the resulting S-parameters (S_11_, S_21_). Thus, the usability of the composites in electromagnetic environments was systematically analyzed. Samples with dimensions of 22 × 10 × 3 mm were used for electromagnetic shielding measurements.

## 3. Results and Discussion

### 3.1. BET (Brunauer, Emmett and Teller) Analysis Results of Activated Carbon

The surface area, pore diameter, and pore volume of the synthesized activated carbon were investigated using BET absorption–desorption isotherms. Table 3 shows the BET surface area examination results.

According to the BET surface area analysis results, the specific surface area of activated carbon was 714.33 m^2^/g, while the Langmuir surface area reached 1069.44m^2^/g. The total pore volume was 0.375 cm^3^/g, and the average pore diameter was in the range of 20.98–32.45 Å. These data reveal the mesoporous structure of activated carbon and demonstrate that the material is effective in terms of both surface interactions and ion transfer [24]. According to t-plot analysis, the micropore area and external surface area were evenly distributed.

Many researchers have highlighted the advantages of the activation process using potassium hydroxide, including the attainment of a high surface area, a fine pore distribution, reduced environmental pollution, and a low cost [25,26,27,28]. Researchers report that during the carbonization process, a series of reactions occur between the activating agent and carbon-rich materials at 400–900 °C, trapping gaseous volatiles such as CO_2_ and CO within the carbon material, creating a highly porous structure. Some of these reactions are as follows. Metallic potassium is produced in reaction number 2 during the activation process. It is assumed that the produced metallic potassium penetrates the internal structure of the carbon matrix, creating new pores. Since the -OH released from KOH during these processes can become trapped in the pores formed, an increase in the porosity and oxygen content of the final activated carbon materials can also be observed [26,29,30,31,32].

6KOH + 2C → 2K + 3H_2_ + 2K_2_CO_3_ (2)

K_2_CO_3_ + 2C → K_2_O + 2CO (3)

K_2_CO_3_ → K_2_O + CO_2_ (4)

2K + CO_2_ → K_2_O + CO (5)

In the study of Tseng et al. on the production of activated carbon from corncob, based on the continuity in various physical properties of chemically activated carbons with different ratios of KOH/corncob, two competitive reaction pathways for pore development were proposed to dominate the activation process: physical activation and etching with KOH. Very low and high BET surface area values per unit mass of raw material were found to be 46 and 435 m^2^/g for the former and the latter methods, respectively [33]. Divya et al. reported that the BET surface analysis values of the activated carbons obtained using phosphoric acid varied between 486–806 m^2^/g [34]. In some studies, BET surface area can reach values such as 2000–3000 m^2^/g [35,36,37]. In this context, the results obtained are moderate and satisfactory, and applicable for activated carbon production.

### 3.2. Electrical Conductivity Measurement of Activated Carbon

Table 4 presents the electrical conductivity values for the activated carbon sample based on experimental data. These measurements are important for evaluating the conductivity performance of the sample and determining its potential in composite applications.

In electrical conductivity tests, the value measured with a four-point probe was determined to be 69.5 S/m. This value is at a conductivity level suitable for EMI shielding applications, demonstrating the synergistic contribution of the carbon structure. With these properties, activated carbon derived from buckwheat hulls supports a sustainable approach and exhibits functional properties capable of suppressing EMI. Conductivity values of activated carbon vary widely depending on factors such as production method, structural properties, and porosity.

Graphite, a well-known allotrope of carbon, generally has an electrical conductivity ranging from 10^3^ to 10^4^ S/cm. Single-layer graphene has a very high conductivity, reported at 10^4^ to 10^5^ S/cm [38]. The conductivity of carbon nanotubes (CNTs) has been reported in the range of 10^2^ to 10^4^ S/cm, depending on their structural properties [39]. In a review study by Barroso on the electrical conductivity of activated carbon, the conductivity values of activated carbons obtained from pine trees and coconuts were given as approximately 0.1 and 1.2 S/cm, respectively, depending on the amount of carbon contained in the activated carbon [40]. While the conductivity of activated carbon is lower than that of graphite, graphene, and carbon nanotubes, it is approximately 58 times better than that of activated carbons derived from pine trees and coconuts. Thus, it offers a reasonable level of conductivity for polymer matrix composites. The lower electrical conductivity of activated carbon compared to carbon nanotubes and graphene reflects the conductivity limitations inherent in activated carbon’s amorphous and porous structure.

### 3.3. XRD and SEM Analysis of Activated Carbon

**Figure 3:**
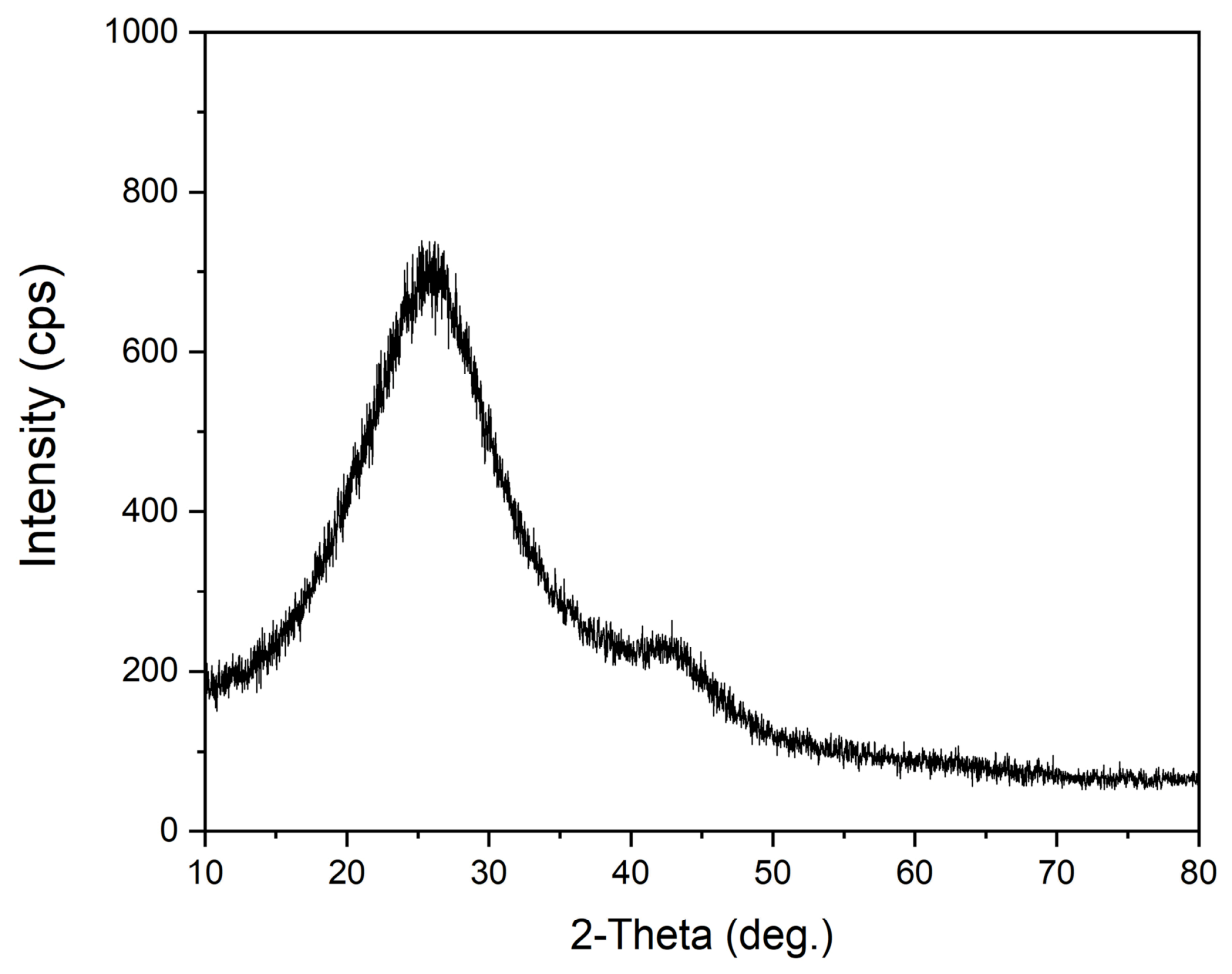
XRD spectrum of the activated carbon sample.

XRD analysis was applied to determine the structural properties of the activated carbon. As shown in Figure 3, the diffractogram of the activated carbon exhibits a single broad, low-intensity diffraction band centered at 2θ =~24°, which indicates that the sample is largely amorphous. This band corresponds to the reflection of disordered carbon and reflects the poor graphitic order of the material, i.e., the random orientation of small, imperfectly stacked graphene-like layers that is characteristic of activated carbons. No sharp crystalline reflections (e.g., those of graphitic carbon or residual potassium compounds) are observed, confirming the absence of a well-developed crystalline carbon phase and of residual inorganic impurities.

This pattern is fully consistent with the XRD behavior reported for activated carbons in the literature: for carbons of this type, the diffraction maximum at 2θ ≈ 25° and the overlapping 100/101 maxima at 2θ ≈ 42° typically merge into a broad, diffuse profile spanning this angular range [41,42,43]. Comparable results have been reported for activated carbons prepared by different activation routes and from different biomass precursors: H_3_PO_4_-activated carbons from jujube stones [41], ZnCl_2_-activated carbons derived from orange peel [43], and steam-activated carbons from eucalyptus wood chips [42] all display the characteristic broad band of amorphous carbon. A systematic investigation of activated carbons prepared under various activation conditions likewise reports two broad diffuse bands centered at 2θ ≈ 23° and 43°, assigned to the 002 and 100/101 sets of planes of fully disordered graphene layers [44]. Consequently, when the XRD profile of the activated carbon obtained in this study is evaluated against these literature reports, it is seen to be in good agreement with the results of similar studies [41,42,43,45].

**Figure 4:**
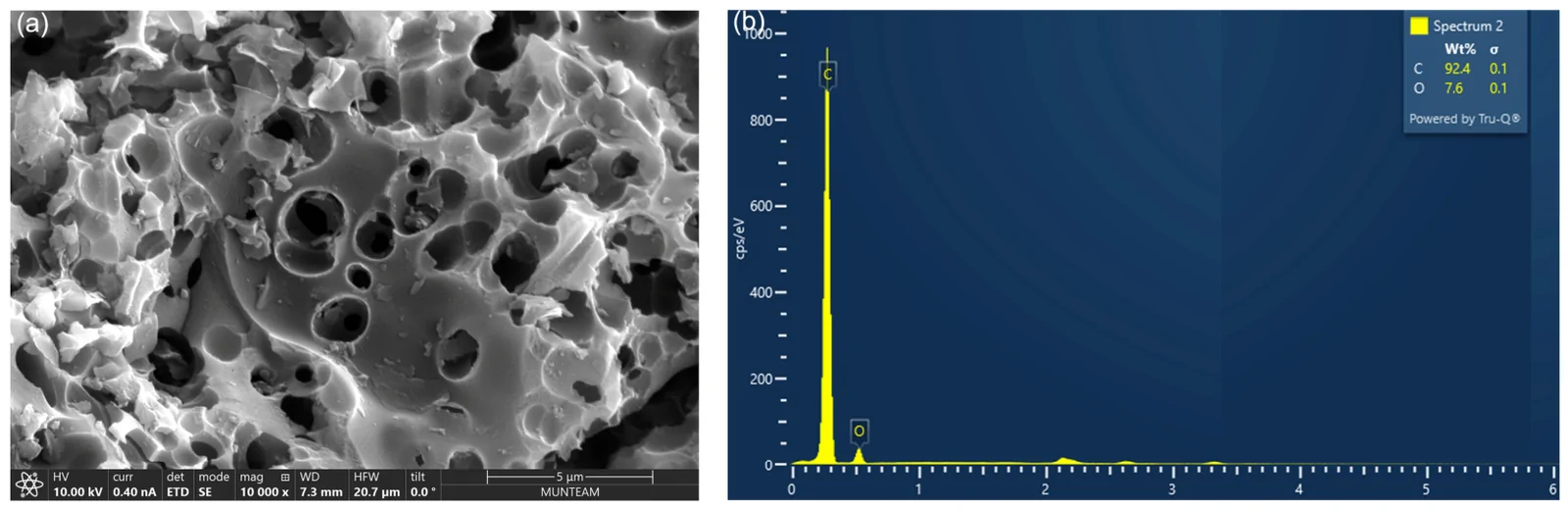
SEM image (**a**) and overall EDS spectrum (**b**) of the activated carbon, revealing a porous sponge-like morphology and a carbon-dominated composition with trace oxygen. The Au/Pd signals arise from the conductive sputter coating used for SEM imaging and were excluded from the compositional analysis.

Scanning electron microscopy analysis enabled high-resolution examination of the surface morphology and pore structure of the activated carbon. EDS analysis, performed to determine the elemental composition of the surface, revealed that the sample contained a large proportion of carbon and also contained trace amounts of other elements such as oxygen. Figure 4 shows the SEM/EDS analysis results of activated carbons produced by activating them with a 1:0.5 ratio of KOH. The high surface area and porous structure of the activated carbon are evident in the SEM image. EDS analyses provided information on the chemical composition of the synthesized activated carbon. EDS analysis, given in Figure 4b, reveals that the carbon content of the activated carbon with a 1:0.5 ratio of buckwheat hulls to KOH reached 92.4%. Impurities such as K and Ca were not observed in the EDS analysis of the activated carbon.

**Figure 5:**
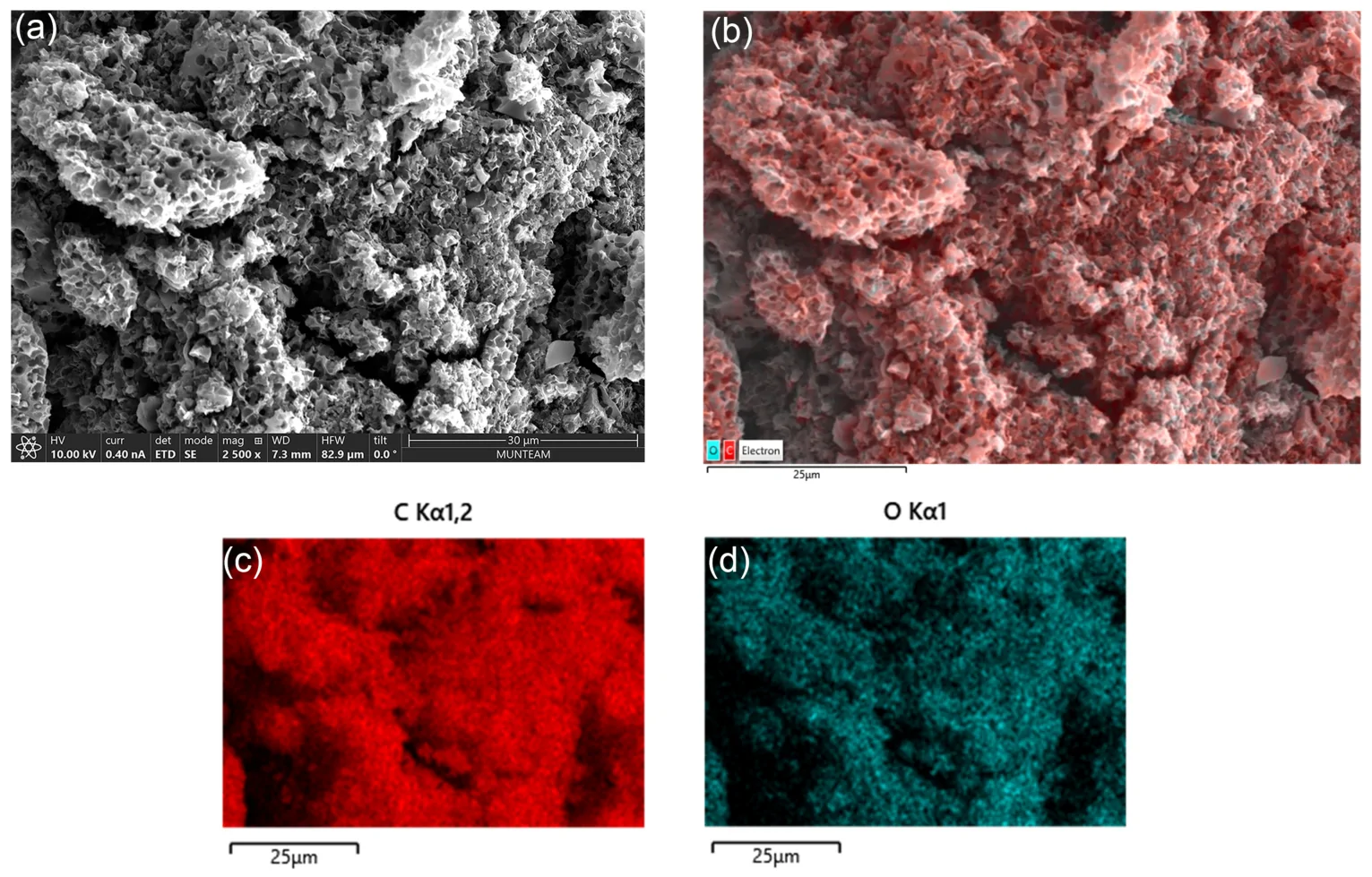
(**a**) SEM image of the activated carbon (**b**) Element-distribution map showing the pixels indexed as carbon (red) and oxygen (cyan), separated elemental distribution maps for C (**c**) and O (**d**). The quantitative elemental composition of the activated carbon was obtained by overall (area-scan) EDS and is presented in Figure 4 (C: 92.4 wt%; O: 7.6 wt%); Au/Pd signals from the sputter coating were excluded from the quantification.

The SEM image of the activated carbon (Figure 5a) is included alongside the corresponding elemental distribution maps (Figure 5b–d) for direct comparison; the morphology has already been described in Figure 4a. The EDS elemental maps (Figure 5b–d) reveal that carbon and oxygen are distributed homogeneously over the analyzed area, indicating a uniform surface composition of the activated carbon.

SEM analysis of KOH-activated buckwheat hull-derived activated carbon revealed a highly porous, irregular, and microscopically fragmented surface structure. The SEM image demonstrates that the activated carbon has acquired an improved surface morphology following pyrolysis and chemical activation. This type of surface morphology is particularly advantageous for electromagnetic wave interaction, ion transport, and surface adsorption processes. The porous structure increases the surface area of materials and supports both electrical and dielectric properties. As previously explained, overall EDS quantification showed a carbon-dominated composition (C: 92.4 wt%; O: 7.6 wt%), with the Au/Pd peaks originating solely from the conductive sputter coating (Figure 4b). This high carbon content demonstrates that pretreatment, pyrolysis conditions, and KOH activation were effective. In addition, it has been reported in the literature that EDS analyses of Fe_3_O_4_ particles obtained from similar solution-based syntheses reveal mainly Fe and O elements, with a surface Fe:O ratio of approximately 3:4 [46,47]. Furthermore, the absence of elemental impurities such as K and Ca in the analysis is significant, as it demonstrates that residues that could negatively impact performance are avoided, particularly in applications such as energy storage and EMI shielding.

Similar surface morphologies and high carbon contents have been reported in the literature [39,48] for activated carbons obtained by activating different biomasses with KOH under similar conditions. For example, SEM analyses of activated carbons obtained from lignocellulosic wastes such as rice hulls, walnut shells, hazelnut shells, and palm kernels have revealed dense microporous structures, which have been shown to provide advantages in terms of adsorption, electrochemical capacity, and EMI attenuation [39,48].

Activation with KOH creates pores in the carbon skeleton through both physical and chemical means, significantly increasing the surface area of the material. However, when the amount of KOH is excessive, some studies in the literature have reported that K or potassium-based residues remain on the surface, which can reduce conductivity and negatively affect application performance. However, it is also reported that these residues can be removed with an intense acid washing process [49]. In this context, the buckwheat hull/KOH 1:0.5 ratio and acid washing process used in this study provided optimum porosity as well as obtaining a pure carbon structure without leaving any chemical residue on the surface.

### 3.4. XRD and Electron Microscopy Analysis Results of Fe_3_O_4_ Particles

**Figure 6:**
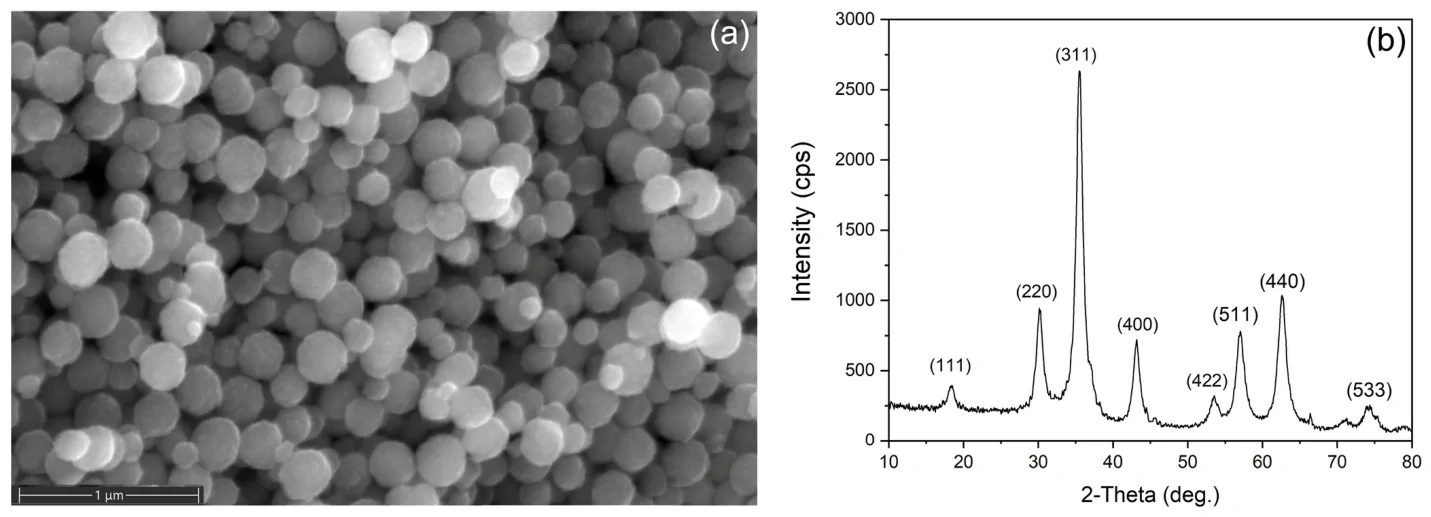
(**a**) SEM micrograph and (**b**) XRD analysis of Fe_3_O_4_ particles.

Electron microscopy (FE-SEM) analysis was performed to examine the surface morphology, particle size, and agglomeration of the Fe_3_O_4_ particles. Figure 6a,b reveal the SEM micrograph and XRD analysis of Fe_3_O_4_ particles, respectively. A high-magnification micrograph shows that the majority of the particles are spherical or semi-spherical, with a tendency to agglomerate in some areas.

Fe_3_O_4_, being a soft magnetic material, plays a particularly effective role in the absorption of low-frequency electromagnetic waves. The high surface area and interparticle interfaces created by particles allow electromagnetic waves to undergo multiple scattering and reflection, facilitating the absorption of wave energy. Furthermore, this morphology enables more efficient loss mechanisms, such as hysteresis loss, loop current loss, and magnetic resonance.

This phenomenon is attributed to dipole–dipole interactions between the particles due to the magnetic properties of Fe_3_O_4_, and has been described similarly in the literature [50]. The tendency to agglomerate is a common structural feature, particularly for magnetic particles synthesized using solution-based methods, and highlights the importance of surface modification in final applications.

The functions of the materials used during the production of Fe_3_O_4_ magnetite particles by the solvothermal method are as follows: Sodium citrate provides a ligand/stabilizer and a reducing agent. Sodium acetate provides a basic environment and a hydroxyl source, as well as the formation of complex/hydroxide intermediates. Finally, ethylene glycol acts as a high-boiling solvent during heating and reduction. The reactions occurring during this process are given in the literature as follows:

FeCl_3_⋅6H_2_O ⟶ Fe^3+^ + 3Cl^−^ + 6H_2_O (6)

2Fe^3+^ + Fe^2+^ + 8OH^−^ ⟶ Fe_3_O_4_ + 4H_2_O (7)

FeCl_3_⋅6H_2_O + Na_3_C_6_H_5_O_7_ + NaOAc + EG → Fe_3_O_4_(s) + by-products (8)

The production conditions, morphologies, and sizes (200–300 nm) of the Fe_3_O_4_ particles obtained in this study largely coincide with those produced in similar studies in the literature [51,52,53].

The XRD pattern of the synthesized magnetic nanoparticles is illustrated in Figure 5b. The diffraction peaks observed at 2θ values of approximately 30.15, 35.5, 43.1, 53.4, 57.0, and 62.6 are indexed to the (220), (311), (400), (422), (511), (440), (533) crystal planes, respectively [52,53]. These characteristic peaks are in excellent agreement with the standard JCPDS data for magnetite (JCPDS No. 19-0629), confirming the formation of a face-centered cubic (FCC) inverse spinel structure [54]. The high intensity and narrow width of the diffraction peaks, particularly the dominant reflection, indicate a high degree of crystallinity and the successful synthesis of nanoparticles [55]. The absence of significant impurity peaks, such as those corresponding to hematite or goethite, further validates the phase purity of the magnetite [54].

### 3.5. Morphology of Hybrid Composites

**Figure 7:**
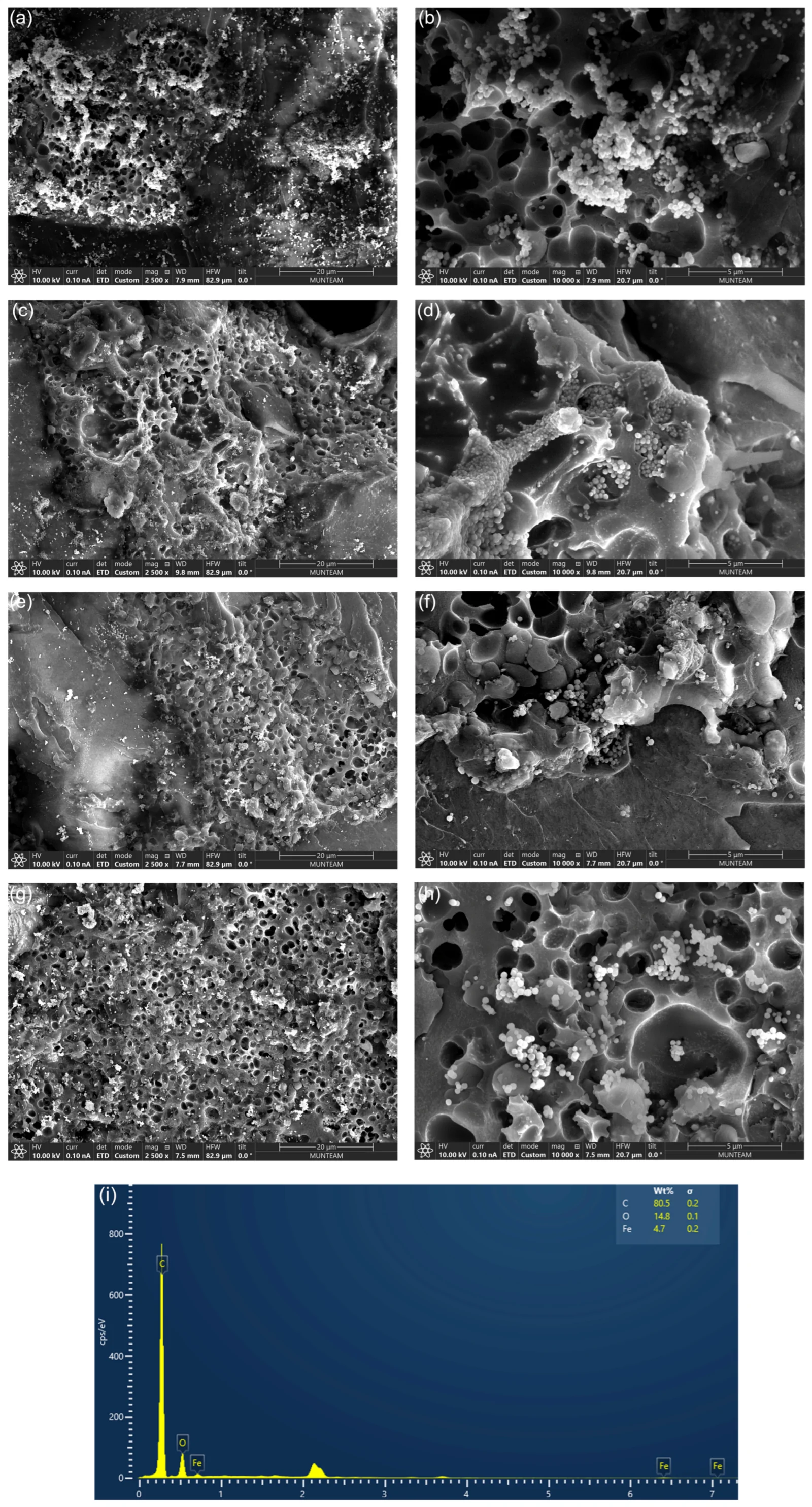
Low- and high-magnification SEM micrographs of hybrid composites: (**a**,**b**) Nov1-15, (**c**,**d**) Nov3-15, (**e**,**f**) Nov5-15, and (**g**,**h**) Nov7-15. The left-hand column shows the overall surface morphology at low magnification, and the right-hand column shows the corresponding high-magnification detail. (**i**) Overall EDS spectrum acquired from the Nov7-15 composite.

In this study, SEM micrographs of the hybrid composites were obtained to gain important insights into the surface morphology and the interaction between the magnetic phase and the carbon matrix. Low- and high-magnification SEM micrographs of the fabricated hybrid composite samples showing the distribution of activated carbon and Fe_3_O_4_ particles within the Novolac resin matrix are shown in Figure 7. The SEM micrographs were taken by preparing the fractured surfaces of the composites. While the Novolac matrix structure in the micrographs of the hybrid composites exhibited a homogeneous and dense surface, the porous structure of the activated carbon and the distribution of Fe_3_O_4_ particles within the matrix in the hybrid composites were observed at different rates.

Figure 7a,b shows low- and high-magnification SEM micrographs of the Nov1-15 hybrid composite, which contains 1 wt.% of activated carbon and 15 wt.% of Fe_3_O_4_. The micrographs clearly show a dense concentration of Fe_3_O_4_ particles with a spherical morphology within the Novolac matrix. At the same time, the porous structure of the activated carbon is clearly visible in the micrograph at high magnification. It is noteworthy that Fe_3_O_4_ particles tend to cluster in some areas and exhibit a more homogeneous distribution in others. In the micrographs shown in Figure 7a, Figure 7c, Figure 7e, and Figure 7g at low magnifications, corresponding to Nov1-15, 3-15, 5-15, and 7-15, respectively, it can be observed that the presence of activated carbon in the microstructure increases with its weight percentage. Composites with higher activated carbon content exhibit more prominent carbon-rich regions, providing clear microstructural evidence supporting the compositional differences. A microstructure photograph of composites containing 3% activated carbon at 10kX magnification shows that Fe_3_O_4_ nanoparticles in the submicron size range generally appear as clusters embedded in carbon-rich folds or as individual spheres (Figure 7e).

The high-magnification micrographs (Figure 7f) of the 5% activated carbon fraction appear to be well-integrated, providing a secondary carbonaceous network that may enhance the overall connectivity of the composite [56]. The Novolac matrix acts as a continuous binder, effectively encapsulating the Fe_3_O_4_ spheres and activated carbon particles. The synergy between the high-surface-area activated carbon and the magnetic Fe_3_O_4_ spheres within the phenolic matrix is particularly advantageous for functional applications. In this hybrid system, the AC provides dielectric loss pathways, while the magnetite spheres contribute magnetic loss, potentially leading to enhanced electromagnetic interference shielding or microwave absorption properties [57].

The high-magnification SEM image of the hybrid composite, sample code Nov-7-15, containing 7% activated carbon and 15 wt.% Fe_3_O_4_ filler material, is presented in Figure 7h. The distribution of spherical Fe_3_O_4_ particles is clearly observed within the composite structure, and with increasing activated carbon content, denser pore formations appear on the surface. Large pores and a tendency for particles to cluster are particularly noticeable in some areas of the structure. The increase in activated carbon content significantly altered the morphology of the composite structure, leading to a predominance of porous regions. This confirmed the integration of the hybrid fillers into the composite structure, while also disrupting homogeneity and increasing microstructural diversity. The findings indicate that, as seen in Figure 7h, increasing the amount of activated carbon significantly increases porosity in the composite structure. The EDS analysis of the Nov7-15 composite (Figure 7i) revealed a composition of 80.5 wt.% C, 14.8 wt.% O, and 4.7 wt.% Fe, confirming the presence of the magnetite-derived iron within the carbonaceous structure. Although additional hybrid composites containing a constant 5 wt.% activated carbon and varying magnetite contents (10, 15, 30, and 45 wt.%) (Table 2) were also analyzed; their SEM micrographs were not included here for brevity. The microstructural features observed in these composites were found to be consistent with those discussed for the presented compositions, showing similar morphological characteristics and nanoparticle distributions.

### 3.6. Thermal Analysis (DTA/TGA) Results of Hybrid Composites

Differential Thermal Analysis (DTA) is a technique used to detect endothermic and exothermic changes that occur during heating or cooling of a material. This method provides information about the material’s thermal stability, decomposition temperatures, phase transitions, and crystallization behavior. Endothermic peaks observed in DTA curves reflect water loss, melting, or other heat-absorbing processes in the material. Exothermic peaks, on the other hand, are associated with heat-releasing processes such as material degradation, crosslinking reactions, or crystallization.

**Figure 8:**
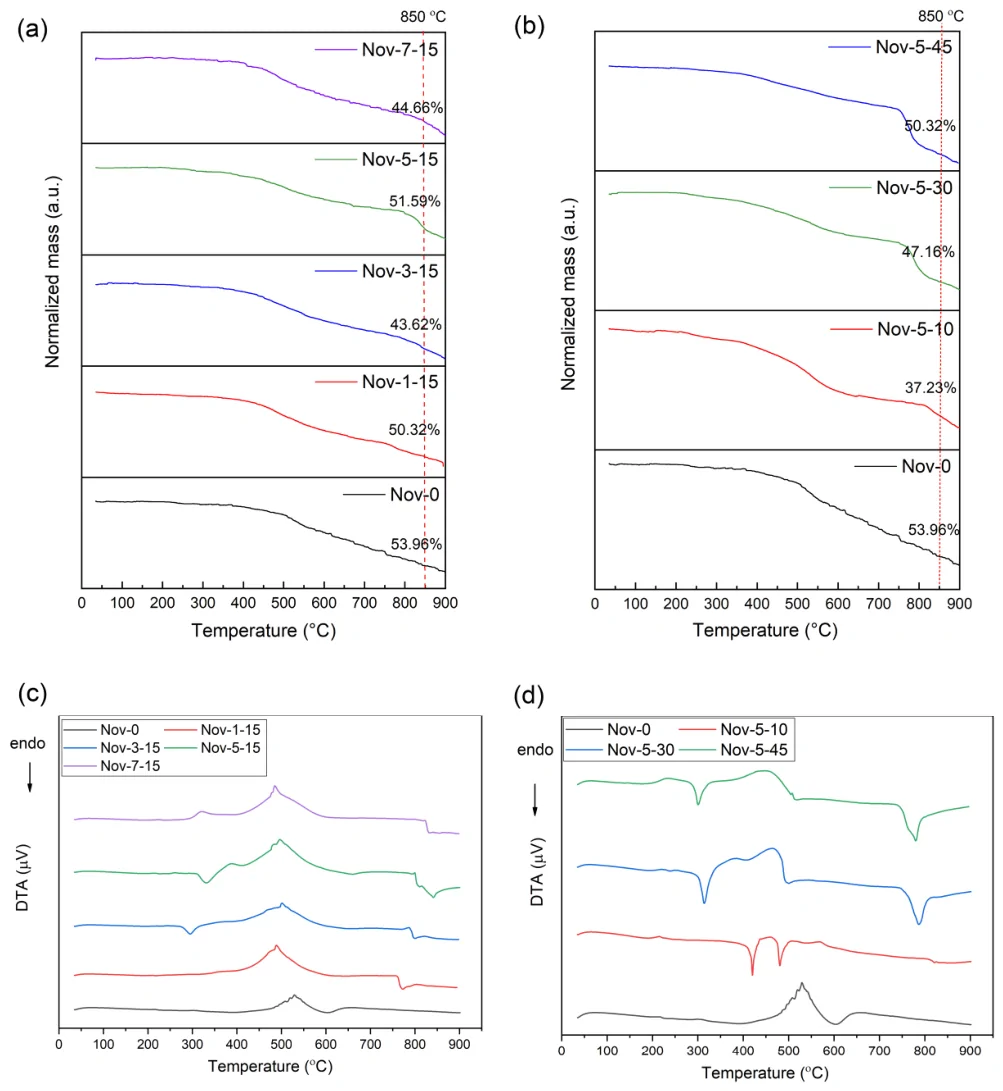
Thermogravimetric (**a**,**b**) and differential thermal (**c**,**d**) analysis curves of novolac-based composite. TGA traces (**a**,**b**) are plotted as normalized mass (a.u.) and offset vertically for clarity; the dashed line at 850 °C marks the temperature at which residual masses are compared.

Figure 8 gives the thermogravimetric analysis (TGA) and differential thermal analysis (DTA) curves of the neat Novolac matrix and the composites containing 15 wt.% magnetite and 1, 3, 5, and 7 wt.% activated carbon (designated Nov-1-15, Nov-3-15, Nov-5-15, and Nov-7-15, respectively) and the composites containing AC/magnetite composites containing 5 wt% activated carbon and 10, 30, and 45 wt% magnetite (designated Nov-5-10, Nov-5-30, Nov-5-45, respectively) recorded from 25 °C to 900 °C under an ambient atmosphere.

In TGA curves, residual mass is normalized to the initial mass at 30 °C. The data from TGA, including the onset degradation temperature at a 2% weight loss (T_on_), the temperature corresponding to 50 wt% weight loss value (T_50%_), and the temperatures of the maximum weight loss (T_max_), as well as the char yield values at 850 °C of the neat Novolac sample and Nov/AC/Fe_3_O_4_ composites, are shown in Table 5.

According to Table 5, except for the Nov-1-15 sample, the addition of the AC/Fe_3_O_4_ filler increased the initial degradation temperature. The comparatively low T_on_ of Nov-1-15 may be tentatively attributed to the very low carbon content (1 wt.% AC) of this composite, for which the dispersed carbon particles may act as local hot spots that promote the early onset of matrix degradation. At higher carbon loadings (~2–3 wt.% AC), the carbon phase appears to bind the Fe_3_O_4_ particles together and to form a continuous barrier network [58]; such a filler network may physically retard the escape of volatile degradation products, contributing to the increase in T_on_ observed for the Nov-3-15, Nov-5-15, and Nov-7-15 samples [59]. The T_50%_ temperatures given in Table 5 are the temperatures at which 50% of the mass loss occurs and provide a direct measure of the stability of composites at medium and high temperatures. The neat Novolac Nov-0 sample reaches its T_50_% value at 819 °C; this temperature increases by 29 °C for Nov-1-15, reaching 848 °C, while for Nov-3-15 it reaches a maximum value of 898 °C (79 °C higher than Nov-0), then drops to 845 °C for Nov-5-15, and finally returns to 882 °C for Nov-7-15. This non-monotonic behavior may reflect the competition between Fe_3_O_4_–AC interfacial contact and the progressive saturation of the proposed interfacial interaction capacity. As the AC fraction increases from 0 to 3 wt.%, the Fe_3_O_4_:AC mass ratio decreases towards 5:1, allowing an increasing fraction of the AC particles to establish intimate contact with the magnetite surface. Magnetite-based and related metal-oxide fillers have previously been reported to catalyze char formation and to improve the compactness of the residual char in polymer matrices [60,61], and metal-oxide–carbon interfacial effects of this type have been proposed as condensed-phase flame-retardancy mechanisms [62]. In the present study, however, direct spectroscopic evidence for Fe–O–C bridging was not obtained, and the interfacial interaction is therefore discussed here as a mechanistic hypothesis. For Nov-5-15, the decrease in the Fe_3_O_4_:AC mass ratio to 3:1 suggests that the AC loading exceeds the interfacial capacity of the magnetite surface; the excess carbon fraction that remains without intimate Fe_3_O_4_ contact may undergo accelerated thermal decomposition in the 700–850 °C range, contributing to the intermediate-temperature mass loss—a “complex, non-linear relationship” also reported for carbon-rich composites above the percolation threshold [63,64]. For Nov-7-15, the higher AC content (7 wt.%) favors direct AC↔AC contacts, which partially restore a thermally stable carbon network; however, the T_50%_ of Nov-7-15 (882 °C) remains below that of Nov-3-15 (898 °C), indicating that the restoration is incomplete because the interfacial contribution is partially saturated.

Table 5 also illustrates the effect of magnetite loading on the thermal stability of AC-containing composites, comparing Nov-5-10, Nov-5-30, and Nov-5-45, which contain a fixed AC content of 5 wt.% and 10, 30, and 45 wt.% Fe_3_O_4_, respectively. T_on_ responds non-monotonically to the Fe_3_O_4_ loading: 232 °C for Nov-5-10 (20 °C below Nov-0), 294 °C for Nov-5-30 (42 °C above Nov-0), and 249 °C for Nov-5-45 (3 °C below Nov-0). A qualitatively similar dependence is observed for T_50%_: 886 °C for Nov-5-30 (67 °C above Nov-0) and 845 °C for Nov-5-45 (26 °C above Nov-0); the T_50%_ of Nov-5-10 could not be quantified from the available TGA data. In Nov-5-45, the high Fe_3_O_4_ loading (45 wt.%) favors Fe_3_O_4_ aggregation [64], so that particle–particle interactions may dominate over the proposed AC–Fe_3_O_4_ interfacial contacts, resulting in a less effective thermal barrier [63]. Consistent with this, the T_50%_ of Nov-5-45 is 41 °C lower than that of Nov-5-30, and T_on_ returns to a value close to that of Nov-0.

The DTA analysis shown in Figure 8c reveals a single exothermic peak for neat Novolac at 529 °C, attributed to the cross-linking of the phenolic matrix. With the addition of Fe_3_O_4_/AC, this exotherm shifts to 489 °C, 501 °C, 496 °C, and 485 °C for Nov-1-15, Nov-3-15, Nov-5-15, and Nov-7-15, respectively, with a non-monotonic dependence on the AC loading. The shift in the principal exotherm to lower temperatures suggests an earlier onset of matrix degradation; the lowest value observed for Nov-7-15 (485 °C) suggests that the AC fraction not in intimate contact with Fe_3_O_4_ promotes this earlier degradation response.

The Nov-7-15 sample, containing 7 wt% AC, demonstrates that unbound AC causes a temperature drop [65]. As can be seen in Figure 8d, Nov-5-30 and Nov-5-45 samples display endothermic peaks at lower temperatures (315–420 °C) and higher temperatures (780–788 °C). The high-temperature endothermic shoulder observed near 780 °C in the Nov-5-30 and Nov-5-45 samples is consistent with the thermal decomposition of activated carbon in air. This process converts the Fe_3_O_4_–AC interface to a more graphitic char [66]. The disappearance of the 530 °C exotherm in the Novolac composites indicates that the Fe_3_O_4_–AC filler network interplays with, rather than simply accelerates, the oxidative chemistry of the matrix [58,67]. The dual endothermic profile of Nov-5-10 at T_max_ = 420/480 °C is mechanically distinct from the 314/788 °C and 302/780 °C pairs exhibited by Nov-5-30 and Nov-5-45. In the phenolic pyrolysis study reported by Torres-Herrador et al. [68], the 420–480 °C range corresponds to the overlap region between dehydration and depolymerization and the breaking/late cross-linking of methylene bridges. The exothermic peak at 420 °C on the DTA curve of Nov-5-10, which we tentatively attribute to the catalytic effect of the Fe_3_O_4_/AC system on matrix degradation, shifts to 314 °C and 302 °C for Nov-5-30 and Nov-5-45, respectively. The high-temperature endotherm appears only for Nov-5-30 and Nov-5-45 (~788/780 °C); its invariance at ~780 °C suggests that Fe_3_O_4_-promoted structural ordering of the carbonaceous char approaches saturation from Nov-5-30 onward. Endothermic events in the 780–800 °C region have been associated with H_2_ release during high-temperature graphitization and with the transformation of the char into a more ordered, hexagonal carbon lattice [58,68], consistent with the well-documented iron-catalyzed graphitization of carbonaceous precursor [69,70]. For the Nov-5-10 sample, this process appears not to have been activated to a comparable extent since this formulation contains 10 wt.% Fe_3_O_4_ but only 5 wt.% AC, it is plausible that the magnetite content is insufficient to establish intimate interfacial contact with the entire AC surface through the proposed Fe–O–C interaction. Direct spectroscopic verification of such interfacial bonding (e.g., by XPS or Raman) was beyond the scope of the present study, and the Fe–O–C anchoring is therefore presented as a mechanistic hypothesis to be examined in future work, as detailed characterization is typically required to confirm such interfacial assumptions in comparable iron–carbon systems [71]. The char residue of the neat Nov-0 resin is 46.04%. In the Nov-X-15 series, increasing the AC content at a constant Fe_3_O_4_ loading of 15 wt.% yields the same non-monotonic, M-shaped response previously documented for T_50%_ and T_on_. The variation in the char residue at 850 °C is consistent with a percolation-limited Fe_3_O_4_–AC interfacial contribution: Nov-3-15 (Fe_3_O_4_:AC ≈ 5:1) exhibits the highest char retention (56.38%), suggesting that the magnetite surface most effectively stabilizes the char at this ratio through the proposed interfacial interaction. Within the Nov-5-X series, the char yield decreases from 62.77% (Nov-5-10) to 52.84% (Nov-5-30) and 49.68% (Nov-5-45), indicating that the increasing Fe_3_O_4_ fraction contributes progressively less to the residual mass than the carbon phase. Notably, the activated carbon-rich samples Nov-3-15 (56.38%) and Nov-7-15 (55.34%) still outperform Nov-5-30 (52.84%), indicating that the AC content is more effective than additional Fe_3_O_4_ in promoting the residual char.

### 3.7. Limiting Oxygen Index (LOI) Analysis Results of Hybrid Composites

LOI (Limiting Oxygen Index) analysis is a widely used test method to determine the flammability of a material. This analysis measures the minimum percentage (%) of oxygen required for the material to sustain combustion. Higher LOI values indicate a material’s flame-retardant properties, while lower values indicate higher flammability. The analysis results provide direct information about the fire safety performance of the material and, when evaluated together with other thermal analysis results (TGA, DTA), offer a comprehensive view of the material’s overall behavior in response to heat and flame impact [19,21,72,73]. For the hybrid composite samples, the mixing ratios of which are given in Table 1 and Table 2, the LOI values were calculated from the char yields at 850 °C (i.e., char weight/initial sample weight) according to Equation 1. Since this equation is not an independent measurement, it inherits every trend of the high-temperature char residue; any difference between the measured and calculated LOI values can therefore only be attributed to mechanisms that act on the condensed-phase combustion response without altering the mass retention at 850 °C. In addition, to compare the calculated values with the measurements, only the samples from Table 1 were tested using a Concept-brand LOI tester in accordance with ASTM D 2863 [22] and ISO 4589-2 [23] standards; the results are summarized in Table 6.

From Table 6, the measured LOI values of all the tested samples (Nov-0 and the four Nov-X-15 composites) are above 30%, indicating that the samples require a much higher oxygen concentration in the environment than the 21% of normal atmospheric conditions in order to sustain combustion. The measured LOI values for Nov-1-15, Nov-3-15, Nov-5-15, and Nov-7-15 are at or slightly below the reference value of 34.31% for the neat Novolac resin, with deviations of −1.21, −0.60, −2.00, and −0.80 percentage points, respectively. The LOI spread across the four composites is only 1.40 percentage points, which is well within the ~2% difference previously identified in the phenolic-thermoset literature [72,73] as the operationally meaningful threshold for the LOI test. Consistent with the DTA observations, the principal 529 °C exotherm of Nov-0 is replaced across the composites by endothermic T_max_ values in the 485–501 °C range; this earlier and broader release of phenolic volatiles supplies additional low-molecular-weight fuel vapor during the LOI burn, lowering the measured LOI despite the high residual char. Since the measured LOI of the Nov-5-X series samples (Nov-5-10, Nov-5-30 and Nov-5-45) could not be determined, this comparison is limited to the five samples of the Nov-X-15 series, for which the measured values were obtained with a Concept-brand LOI tester in accordance with ASTM D 2863 and ISO 4589-2.

The calculated LOI values peak at 40.05% for Nov-3-15, then decrease to 36.86% for Nov-5-15 before rising again to 39.63% for Nov-7-15. Within the proposed Fe–O–C anchoring framework, this pattern can be understood as follows: at Nov-3-15 (Fe_3_O_4_:AC ≈ 5:1) the interfacial contribution is most effective; at Nov-5-15 part of the AC population remains outside the interfacial contact region; and at Nov-7-15 direct AC↔AC contacts begin to supplement the partially saturated interfacial capacity, with the AC transitioning from an active interfacial participant to a self-supporting carbon-network constituent.

The differences between the measured and calculated LOI values (measured minus calculated) are −1.60, −4.27, −6.34, −4.55, and −6.12 percentage points for Nov-0, Nov-1-15, Nov-3-15, Nov-5-15, and Nov-7-15, respectively. Equation 1 systematically overestimates the measured LOI, and the overestimation is most pronounced where the R_850 °C_ value peaks (Nov-3-15 and Nov-7-15), i.e., where char retention is highest but the char quality—the dense, uniform, oxygen-resistant residue implicitly assumed by the equation—is only partially achieved.

For the Nov-5-X series at a constant 5 wt.% AC, the calculated LOI decreases monotonically from 42.60% (Nov-5-10) to 38.63% (Nov-5-30) and 37.37% (Nov-5-45). Within the proposed framework, this decrease is consistent with the saturation of the Fe–O–C anchoring capacity: beyond a critical Fe_3_O_4_ loading—the level at which the proposed Fe–O–C interfacial anchoring appears to saturate—the calculated quantity solely reflects the 850 °C char yield, whereas the measured flame-retardancy response is governed by the joint contribution of the earlier and broader release of volatile species from the phenolic matrix (reflected in the shift in the principal thermal event from 529 °C to the 485–501 °C range in the DTA curves) and of the structural features of the hybrid composites evidenced in the SEM micrographs (Figure 7), as documented in our thermal-analysis and SEM results. As discussed in Section 3.6, the Fe–O–C anchoring is proposed as a mechanistic hypothesis based on the thermal, charring, and flame-retardancy evidence, and was not directly verified by spectroscopy (e.g., XPS or Raman) in the present study; its direct verification is planned as future work, in line with the detailed characterization required to confirm interfacial assumptions in comparable iron–carbon systems [71].

### 3.8. EMI Shielding Properties of Hybrid Composites

In this study, the scattering parameters (S11 and S21) in the frequency range of 8.2–12.4 GHz (X-band) were recorded to calculate EMI SE (SE_T_), reflection loss (SE_R_), and absorption loss (SE_A_) using the following Equations (9)–(11) [1,74,75].

(9) $${SE}_{R}\left(dB\right)=-10log\left(1-{\left|S_{11}\right|}^{2}\right)$$

(10) $${SE}_{A}\left(dB\right)=-10log\left(\frac{{\left|S_{11}\right|}^{2}}{1-{\left|S_{21}\right|}^{2}}\right)$$

(11) $${SE}_{T}={SE}_{R}+{SE}_{A}=20\;log(S_{21})$$

**Figure 9:**
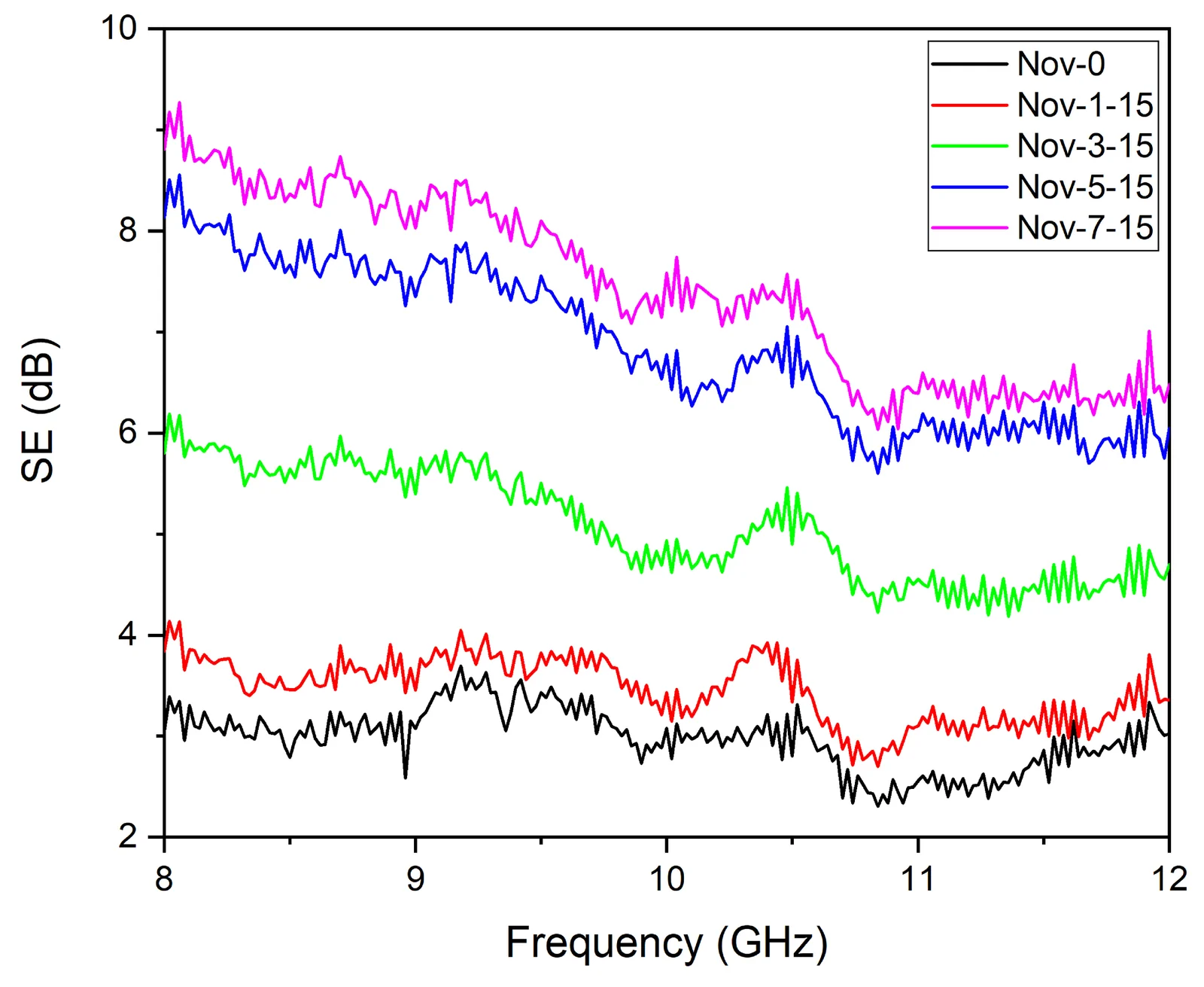
Variation in shielding effectiveness (SE) over the X-band frequency range (8–12 GHz) of Nov-0, Nov-1-15, Nov-3-15, Nov-5-15, and Nov-7-15 samples.

**Figure 10:**
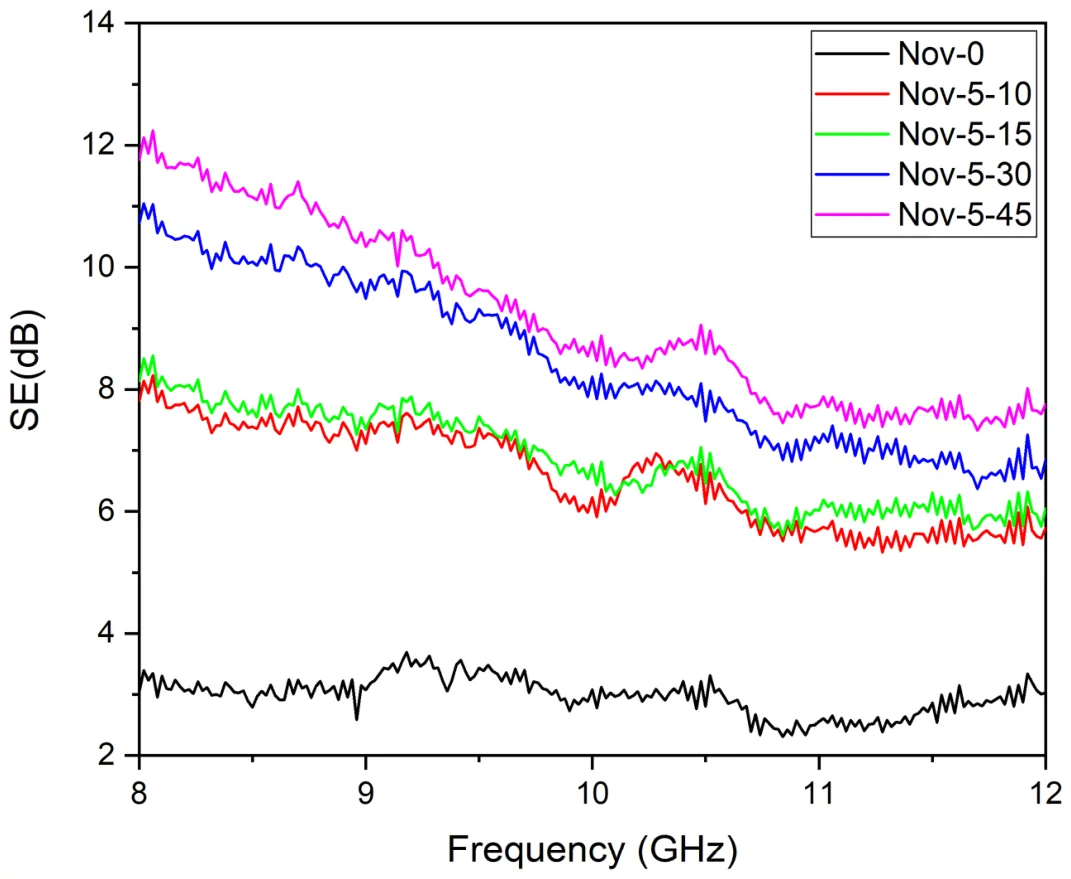
Variation in shielding effectiveness (SE) over X-band frequency range (8–12 GHz) of Nov-0, Nov-5-10, Nov-5-15, Nov-5-30, and Nov-5-45 samples.

The results of measurements of the EMI shielding effectiveness of neat Novolac and hybrid composites in the frequency range of 8 to 12 GHz are shown in Figure 9 and Figure 10. In addition, Table 7 is given with standard deviations in order to see the contributions/effects of SE_T_ and its constituent SE_A_ and SE_R_ values for each sample.

The neat Novolac exhibits negligible shielding capability, with SE values hovering around 2.5–3.5 dB, which suggests that the polymer matrix is almost transparent to electromagnetic waves. A systematic increase in SE_R_ values was generally observed with the addition of activated carbon, and in SE_A_ values with increasing Fe_3_O_4_ addition. In the Nov-1-15, Nov-3-15, Nov-5-15, and Nov-7-15 samples, where the Fe_3_O_4_ amount was kept constant at 15%, and the activated carbon amount was changed from 1 to 7%, an increase in SE_T_ values from 3.46 to 7.43 dB was recorded. The transition from Nov-1-15 (~4 dB) to Nov-7-15 (~8 dB) highlights the critical role of the activated carbon (AC). Increasing AC content enhances the conductivity of the composite, promoting conduction loss and interfacial polarization [59]. Examining Figure 9 and Figure 10, a partial decrease in SE values is observed in all samples with increasing frequency. This decrease is particularly evident in the 10–12 GHz range. The frequency at which the samples exhibit the highest SE values is around 8 GHz. The observed frequency-dependent decline in SE across the X-band indicates that the shielding mechanism is dominated by absorption and interfacial polarization rather than simple surface reflection.

A detailed examination of the SE_A_ and SE_R_ values that make up the SE_T_ value reveals that the expected upward trend in SE_R_ values with an increase in the activated carbon amount was disrupted only in the Nov-7-15 sample (Table 7). While the SE_R_ value of the Nov-5-15 sample was 4.56 dB, the SE_R_ value of the Nov-7-15 sample was 4.22 dB. When the activated carbon content was increased to 7%, the performance increase was limited, and the hybrid composite trend in terms of SE_R_ reached saturation. This indicates that high amounts of activated carbon can cause agglomeration, disrupting the homogeneous distribution, thus limiting the expected increase in shielding effectiveness.

Due to the limited amount of active carbon in SE_R_ for EMI shielding measurements, a second group of hybrid composite samples was produced and analyzed. In these samples, the active carbon ratio was kept constant at 5%, while the Fe_3_O_4_ ratio was increased from 10% to 45%. As shown in Figure 10, as Fe_3_O_4_ loading increases from 10 wt% to 45 wt%, there is a monotonic improvement in SE. The Nov-5-45 sample achieves the highest performance in this series, peaking at approximately 12 dB at 8 GHz. Specifically, the Nov-5-45 sample exhibited higher shielding effectiveness than the others across the entire frequency range, reaching average SET values of 8.77–12.24 dB in the 8–10 GHz range. This demonstrates that the Fe_3_O_4_ filler contributes significantly to the attenuation of electromagnetic waves through magnetic losses. All samples show a slight negative slope (decreasing SE with increasing frequency). This trend is typical for composites where the magnetic loss component (μ”) from Fe_3_O_4_ contributes significantly at lower X-band frequencies, while dielectric losses remain relatively constant [59]. Compared to other magnetite-doped thermoset systems that avoid expensive nanofillers such as carbon nanotubes or MXenes [66], the current Fe_3_O_4_/activated-carbon system offers competitive entry-level shielding performance, although the absolute SE_T_ values (up to 9.19 dB average for Nov-5-45; Table 7) remain below the ~30 dB level regarded as the requirement for commercial shielding applications [3]. Notably, Zhang et al. [76] reported an equivalent EMI shielding effectiveness (SE) of approximately 13 dB for multifunctional Fe_3_O_4_/MWCNT/PMMA foams with a 7 wt% hybrid loading. In this case, attenuation was driven by improved impedance matching and multiple reflections within the microcellular structure. The ability of the current system to achieve almost the same level of attenuation using cost-effective activated carbon instead of specialized multi-walled carbon nanotubes demonstrates the structural efficiency of the Fe_3_O_4_/AC hybrids within the dense novolac matrix [76]. Table 8 shows a comparison of the present Fe_3_O_4_/AC/novolac hybrids with representative EMI shielding systems reported in the literature.

It should be noted that the activated carbon used in this study was derived from buckwheat husk, an agricultural waste, via KOH activation followed by acid washing. The resulting AC exhibits a four-point-probe electrical conductivity of 69.5 S/m, a value that is suitable for EMI shielding applications yet remains considerably lower than that of commercial graphitic fillers such as carbon nanotubes or graphene. Biomass-derived activated carbons are inherently less graphitic and retain oxygen functionalities and structural disorder; indeed, raw unmodified biochar has been reported to provide only 1.5–4 dB of shielding effectiveness, and meaningful attenuation is typically achieved only after modification with highly conductive carbons [77]. Accordingly, the absolute SE_T_ values of the present composites should be evaluated in the context of (i) the intrinsically lower conductivity of the biomass-derived activated carbon phase relative to commercial carbons, (ii) the deliberately low AC loadings employed (1–7 wt.%), which remain far below the 40 wt.% loadings required for agricultural-waste biochar systems that report high shielding effectiveness (26.5–41.5 dB in the X-band) [78,79], and (iii) the dense novolac matrix, which limits the formation of a percolated conductive network. On a per-unit-carbon-loading basis, the Fe_3_O_4_/AC/novolac hybrids therefore deliver competitive absorption-dominated attenuation while additionally offering the flame-retardant functionality discussed in Section 3.6 and Section 3.7, together with the sustainability advantages of valorizing agricultural waste as a low-cost activated carbon precursor.

Beyond the absolute performance, the present study differs from the existing Fe_3_O_4_/carbon literature in its objective, composition, and mechanistic framework. Most published Fe_3_O_4_/carbon systems are designed for microwave absorption rather than EMI shielding and rely on high filler loadings or elaborate nanostructures—e.g., 50 wt.% of three-dimensional porous Fe_3_O_4_/C flowers in epoxy [80] or monodisperse Fe_3_O_4_/C core–shell nanosheets [81]. To the best of our knowledge, the present work provides the first systematic X-band shielding evaluation of a Fe_3_O_4_/biomass-derived-activated-carbon/novolac hybrid composite, including the decomposition of SE_T_ into its absorption and reflection contributions, at moderate loadings (1–7 wt.% AC, 10–45 wt.% Fe_3_O_4_) in a dense structural thermoset and in the absence of any nanoscale carbon filler. The system is also dual-functional: the same Fe–O–C interfacial interaction proposed for the flame-retardancy behavior (Section 3.6 and Section 3.7) is reflected in the dielectric response; direct XPS evidence for Fe–O–C bonding has been reported for analogous Fe_3_O_4_–carbon nanocomposites [82], although verification of such interfacial bonding in the present system would require dedicated spectroscopic characterization (e.g., XPS or Raman), which is identified as future work.

Relative to highly conductive carbon fillers, a trade-off between absolute shielding and practical viability is evident. CNT- and graphene-based networks can indeed deliver considerably higher SE values—e.g., 38.4–47.5 dB in the X-band at only 1.6 mm for CNT–multilayered graphene core–shell hybrid foams [83], or 30 dB for lightweight graphene foam composites [84]—owing to their high aspect ratio and electrical conductivity. Such levels, however, are achieved with nanostructured fillers with multistep processing, and the SE of carbon-nanomaterial networks is strongly thickness-dependent, with high values generally requiring considerable sample thickness or elaborate porous architectures [85]. Reflection-dominated shielding, typical of highly conductive networks and metal-based shields, is also undesirable in applications where absorption is required [86]. In the present hybrids, magnetite improves impedance matching through magnetic loss, and absorption indeed dominates at the highest magnetite loading (Nov-5-45: SE_A_ = 5.58 dB vs. SE_R_ = 3.61 dB), consistent with the absorption-dominated behavior also reported for ferrite-containing polymer composites [3]. Magnetic particles alone, however, cannot provide effective shielding—ferrite loadings as high as 80 wt.% in a polymer matrix yield only ~3.75 dB (at 1 GHz, 10 mm thickness) [3]—which is why the two-phase Fe_3_O_4_/AC combination is advantageous in the present cost- and sustainability-constrained context.

**Table 8:** Comparison of the EMI shielding performance of the present Fe_3_O_4_/AC/novolac hybrids with representative shielding systems reported in the literature.

System	Filler/Loading	Thickness	Frequency Range	SET (dB)	Ref.
Neat novolac	—	3 mm	X-band	2.97	Present work
Nov-1-15	1 wt% AC + 15 wt% Fe_3_O_4_	3 mm	X-band	3.46	Present work
Nov-3-15	3 wt% AC + 15 wt% Fe_3_O_4_	3 mm	X-band	5.077	Present work
Nov-5-15	5 wt% AC + 15 wt% Fe_3_O_4_	3 mm	X-band	6.86	Present work
Nov-7-15	7 wt% AC + 15 wt% Fe_3_O_4_	3 mm	X-band	7.43	Present work
Nov-5-30	5 wt% AC + 30 wt% Fe_3_O_4_	3 mm	X-band	8.45	Present work
Nov-5-45	5 wt% AC + 45 wt% Fe_3_O_4_	3 mm	X-band	9.19	Present work
Fe_3_O_4_/MWCNT/PMMA foam	7 wt% hybrid	—	—	~13	[76]
Raw (unmodified) biochar	—	0.1–0.5 mm	1–3 GHz	1.5–4	[77]
Biochar + 20 wt% acetylene black	20 wt% AB	—	1–3 GHz	39	[77]
KOH-activated RCOS biochar/epoxy (150 S/m)	40 wt% biochar	—	X-band	41.5	[78]
RCOS biochar/epoxy (700 °C; 95 S/m)	40 wt% biochar	—	X-band	26.5	[79]
Fe_3_O_4_–3D reduced porous carbon (1:2)	Fe_3_O_4_:rPC = 1:2	0.5 mm	X-band	41.285	[82]
CNT–multilayered graphene hybrid foam	CVD-grown foam	1.6 mm	X-band	38.4–47.5	[83]
Graphene/PDMS foam	graphene foam	1.0 mm	X-band	~30	[84]
Ti_3_C_2_T_x_@CNT hybrid buckypaper	49.4 wt% MXene	15 μm	X-band	50.4 (60.5 at 100 μm)	[85]

## 4. Conclusions

In this study, multifunctional hybrid composites combining Fe_3_O_4_ nanoparticles and activated carbon derived from agricultural waste (buckwheat hulls) were successfully fabricated by the hot-press forming method for electromagnetic shielding and fireproofing applications. The findings of this work are summarized point by point:

1. Activated carbon was successfully produced from buckwheat hulls by chemical activation with KOH (1:0.5 mass ratio) followed by pyrolysis at 700 °C for 2 h and purification with 0.5 M HCl. The resulting material exhibited a high specific surface area of 714.33 m^2^/g (Langmuir surface area of 1069.44 m^2^/g), a total pore volume of 0.375 cm^3^/g, and an average pore diameter in the range of 20.98–32.45 Å, confirming its well-developed mesoporous structure.
2. The synthesized Fe_3_O_4_ phase crystallized in the face-centered cubic (fcc) inverse-spinel structure, in excellent agreement with JCPDS No. 19-0629, and no hematite or goethite impurity peaks were detected, confirming phase-pure magnetite.
3. At a fixed 5 wt% AC loading, the thermal stability responded non-monotonically to Fe_3_O_4_ content: the onset temperature was 232 °C for Nov-5-10, reached a maximum of 294 °C for Nov-5-30 (vs. 252 °C for neat Novolac) and declined to 249 °C for Nov-5-45; T_50%_ increased from 819 °C to 886 °C (Nov-5-30) and 845 °C (Nov-5-45). This behavior reflects a dual mechanism: at low AC loadings, the carbon particles act as localized oxidation seeds that accelerate early-stage degradation, whereas above a critical threshold, the carbon network binds the magnetite into a continuous barrier that delays oxygen diffusion.
4. The addition of the Fe_3_O_4_/AC hybrids increased the average SE_T_ of the neat novolac matrix from 2.97 dB to 9.19 ± 1.46 dB (Nov-5-45), with a peak value of ~12 dB at 8 GHz. This improvement arises from the combination of Fe_3_O_4_-induced magnetic loss, which raised SE_A_ from 0.89 to 5.58 dB, and the dielectric contribution of the activated carbon network. In contrast, the SE_R_ component remained at ~4.56 dB and reached saturation at higher AC loadings (4.22 dB at 7 wt% AC), which is attributed to agglomeration of the carbon particles disrupting their homogeneous distribution.

All Fe_3_O_4_/AC/Novolac hybrid composites exhibit inherent flame retardancy, with measured LOI values between 32.31% (Nov-5-15) and 33.71% (Nov-3-15), which are tightly clustered around the 34.31% reference value for neat Novolac—a spread of only 1.40 percentage points, which remains within the ~2% threshold that is operationally meaningful for phenolic thermoset LOI tests. This consistent LOI behavior, taken together with the 850 °C char-yield trends and the 485–501 °C endothermic Tmax shift, suggests a contribution of the Fe–O–C interfacial interaction to the condensed-phase flame-retardancy behavior, alongside the carbon loading.

## Figures and Tables

**Table 1:** Table showing the weight ratios of the first group of composite samples.

Sample Code	Novolac Phenolic Resin wt.%	Activated Carbon wt.%	Fe_3_O_4_ Particles wt.%
Nov-0	100	0	0
Nov-1-15	84	1	15
Nov-3-15	82	3	15
Nov-5-15	80	5	15
Nov-7-15	78	7	15

**Table 2:** Table showing the weight ratios of the second group of composite samples.

Sample Code	Novolac Phenolic Resin wt.%	Activated Carbon wt.%	Fe_3_O_4_ Particles wt.%
Nov-0	100	0	0
Nov-5-10	85	5	10
Nov-5-15	80	5	15
Nov-5-30	65	5	30
Nov-5-45	50	5	45

**Table 3:** BET surface area and pore structure analysis results of activated carbon sample.

Parameter	Value	Parameter	Value
BET Surface Area	714.33 m^2^/g	t-Plot Micropore Volume	0.1557 cm^3^/g
Langmuir Surface Area	1069.44 m^2^/g	BJH Adsorption Pore Volume	0.1853 cm^3^/g
t-Plot Micropore Area	320.31 m^2^/g	BJH Desorption Pore Volume	0.0819 cm^3^/g
t-Plot External Surface Area	394.02 m^2^/g	Average Pore Diameter (Adsorption)	20.98 Å
BJH Adsorption Surface Area	307.33 m^2^/g	Average Pore Diameter (Desorption)	21.05 Å
BJH Desorption Surface Area	101.03 m^2^/g	BJH Average Pore Diameter (Adsorption)	24.11 Å
Total Pore Volume (Adsorption)	0.3747 cm^3^/g	BJH Average Pore Diameter (Desorption)	32.45 Å
Total Pore Volume (Desorption)	0.3759 cm^3^/g		

**Table 4:** Data obtained from the electrical conductivity measurement of the activated carbon sample.

Number	Surface Resistance (Rs) [ohm/sq]	Thickness (t) (cm)	Resistivity (ρ) [Ω·cm]	Conductivity (σ) (S/m)
1	14.761	0.1	1.4761	67.75
2	14.093	0.1	1.4093	70.95
3	14.265	0.1	1.4265	70.12
Average	14.373	0.1	1.4373	69.5

**Table 5:** Thermal analysis data obtained from TGA curves.

Sample	T_on_ (°C)	T_50%_ (°C)	T_max_ (°C)	* R_850 °C_ (%)	Max Weight Loss (%)
Nov-0	252	819	529	46.04	53.96
Nov-1-15	208	848	489	49.68	50.32
Nov-3-15	335	898	501	56.38	43.62
Nov-5-15	269	845	496	48.41	51.59
Nov-7-15	399	882	485	55.34	44.66
Nov-5-10	232	-	420/480	62.77	37.23
Nov-5-30	294	886	314/788	52.84	47.16
Nov-5-45	249	845	302/780	49.68	50.32

* Char residue weight at 850 °C.

**Table 6:** % LOI results of neat Novolac and hybrid composites.

Sample Code	% LOI_Measured_	% LOI_Calculated_
Nov-0	34.31 ± 0.11	35.91
Nov-1-15	33.10 ± 0.11	37.37
Nov-3-15	33.71 ± 0.11	40.05
Nov-5-15	32.31 ± 0.11	36.86
Nov-7-15	33.51 ± 0.11	39.63
Nov-5-10	NA	42.60
Nov-5-30	NA	38.63
Nov-5-45	NA	37.37

**Table 7:** SE_A_, SE_R,_ and SE_T_ values of neat Novolac and hybrid composite samples with their standard deviations.

Sample Code	SE_A_ (dB)	SE_R_ (dB)	SE_T_ (dB)
Nov-0	0.89 ± 0.53	2.08 ± 0.52	2.97 ± 0.3
Nov-1-15	1.01 ± 0.51	2.45 ± 0.53	3.46 ± 0.32
Nov-3-15	1.46 ± 0.91	3.61 ± 0.94	5.077 ± 0.53
Nov-5-15	2.29 ± 1.17	4.56 ± 1.59	6.86 ± 0.78
Nov-7-15	3.21 ± 1.45	4.22 ± 1.87	7.43 ± 0.89
Nov-5-10	2.40 ± 1.16	4.18 ± 1.37	6.56 ± 0.81
Nov-5-30	3.79 ± 1.69	4.66 ± 2.28	8.45 ± 1.36
Nov-5-45	5.58 ± 1.20	3.61 ± 2.10	9.19 ± 1.46

## Data Availability

The original contributions presented in this study are included in the article. Further inquiries can be directed to the corresponding author.
